# Is the combination of exercise therapy and health education more effective than usual medical care in the prevention of non-specific back pain? A systematic review with meta-analysis

**DOI:** 10.1080/07853890.2022.2140453

**Published:** 2022-11-04

**Authors:** Pablo Hernandez-Lucas, Raquel Leirós-Rodríguez, Juan Lopez-Barreiro, José L. García-Soidán

**Affiliations:** a Faculty of Physiotherapy, University of Vigo, Pontevedra, Spain; b SALBIS Research Group. Nursing and Physical Therapy Department, University of León. Astorga Ave, Ponferrada, Spain; c Faculty of Education and Sport Sciences, University of Vigo, Pontevedra, Spain

**Keywords:** Back pain, exercise therapy, health education, primary prevention, secondary prevention

## Abstract

**Background:**

Clinical practice guidelines emphasize the importance of the prevention and treatment of non-specific back pain through exercise therapy and health education. However, it has not yet been confirmed that the combination of exercise plus education is more effective than usual medical care.

**Objective:**

The aim of this study was to determine if the combination of exercise plus education is more effective for the prevention of non-specific back pain than usual medical care.

**Materials and methods:**

A systematic search in PubMed, Scopus, Web of Science and Medline was conducted with the terms Back Pain, Neck Pain, Musculoskeletal Pain, Exercise, Exercise Therapy, Health Education, Cognitive Behavioral Therapy, Primary Prevention, Secondary Prevention and Clinical Trial. The inclusion criteria were: articles published from 2016 to 2021, the intervention included exercise and education, and the sample consisted of non-specific back pain patients.

**Results:**

A total of 4 randomized controlled trials were selected (average PEDro score 6.5 points). The meta-analysis showed statistically significant differences in the pain intensity, standardized mean differences was found to be −0.75 (95% CI = −1.41 to −0.08; *p* = 0.03); and in disability, standardized mean differences was found to be −0.24 (95% CI = −0.38 to −0.1; *p* = 0.001).

**Conclusions:**

Interventions combining exercise and education seem to have a greater preventive effect on non-specific back pain than usual medical care.

**Key messages**
- Exercise therapy and health education combination prevent better non-specific back pain than usual care.
- Combining exercise with educational interventions has a higher improvement on disability and kinesophobia than usual care.

## Introduction

Back pain is a global problem and is a leading contributor to the increasing prevalence of disability over the past 30 years [1]. The most common spinal disorder is non-specific back pain (NBP), as it cannot be attributed to a specific underlying disease such as cancer, infection, ankylosing spondylitis, or other inflammatory or infectious diseases [2]. The prevalence of NBP worldwide is 14%, and it is increasing regardless of age, sex, ethnicity, socioeconomic status and geographic region [3]. Specifically, non-specific low back pain is the second leading cause of medical care demand in developed countries, the third leading cause of surgery and functional disability and the fifth leading cause of hospitalization [4,5].

To avoid these serious socioeconomic problems, it is essential to prevent its progression and limiting consequences, such as loss of functionality or work capacity [6,7].

It is essential to know the risk factors associated with NBP in order to prevent it, with the aim of reducing the serious socioeconomic repercussions caused by NBP [8]. This condition presents a multifactorial approach: sedentary lifestyle [9], obesity [10], lack of muscle strength [11,12], lack of flexibility [13] and psychosocial [14,15] and work-related factors [16,17].

This complicates an accurate diagnosis, the definition of a prognosis and the design of effective interventions that reduce the use of invasive methods (surgical and opioid-based pharmacological interventions) [18–20]. Clinical practice guidelines emphasize the importance of the prevention and treatment of NBP through exercise therapy (ET) (i.e. strengthening and stretching exercises and yoga) and health education (HE) (i.e. ergonomics, self-management techniques, pain neuroscience education and stress reduction techniques) [2,21–23].

Several systematic reviews have examined the benefits of ET in preventing pain, disability, loss of quality of life and kinesophobia related to non-specific low back [24–27] and cervical pain [28–33] and that ET is cost-effective versus usual medical care (UC) in the subacute and chronic treatment of NBP [34]. Regarding HE, a meta-analysis published in 2021 concluded that counselling provides improvements in pain and disability, although the effects may be insufficient as monotherapy for patients with NBP [35]. Moreover, no recent systematic reviews have attempted to determine whether the combination of ET plus HE is more effective for the prevention of NBP than UC. Therefore, the primary objective of the present systematic review and meta-analysis was to determine whether the combination of ET and HE is more effective than UC for the prevention of NBP. In addition, as secondary objectives, the effects on other related variables such as disability and kinesophobia were investigated.

## Materials and methods

### Data sources and searches

This study was prospectively registered on PROSPERO (ID: CRD42022311026) and followed the Preferred Reporting Items for Systematic Reviews and Meta-analyses (PRISMA) [36] reporting guidelines in Exercise, Rehabilitation, Sport medicine and Sports (PERSIST) [37] and the recommendations from the Cochrane Collaboration [38]. The PICOS question was then chosen as follows: P – population (participants with NBP); I – intervention (ET plus HE); C – control (UC); O – outcome (characteristics of pain, disability and kinesophobia); S – study design (randomized controlled trial).

A systematic search of publications was conducted in December 2021 in the following databases: PubMed, Scopus, Web of Science and Medline. The search strategy included different combinations with the following Medical Subject Headings (MeSH) terms: Back Pain, Neck Pain, Musculoskeletal Pain, Exercise, Exercise Therapy, Health Education, Cognitive Behavioral Therapy, Primary Prevention, Secondary Prevention and Clinical Trial. The following word was used as a free term: Prevention. The search strategy according to the focused PICOS question is presented in Table 1.

**Table 1.** Search strategy according to the focused question (PICO).

Database	Search equation
PubMed	“back pain” [MeSH Terms] AND (“exercise” [MeSH Terms] OR “exercise therapy” [MeSH Terms]) “back pain” [MeSH Terms] AND (“Health Education” [MeSH Terms] OR “cognitive behavioral therapy” [MeSH Terms]) “back pain” [MeSH Terms] AND (“primary prevention” [MeSH Terms] OR “secondary prevention” [MeSH Terms]) "back pain" [MeSH Terms] AND "prevention" “neck pain” [MeSH Terms] AND (“exercise” [MeSH Terms] OR “exercise therapy” [MeSH Terms]) “neck pain” [MeSH Terms] AND (“Health Education” [MeSH Terms] OR “cognitive behavioral therapy” [MeSH Terms]) “neck pain” [MeSH Terms] AND (“primary prevention” [MeSH Terms] OR “secondary prevention” [MeSH Terms]) "neck pain" [MeSH Terms] AND "prevention" “musculoskeletal pain” [MeSH Terms] AND (“exercise” [MeSH Terms] OR “exercise therapy” [MeSH Terms]) “musculoskeletal pain” [MeSH Terms] AND (“Health Education” [MeSH Terms] OR “cognitive behavioral therapy” [MeSH Terms]) “musculoskeletal pain” [MeSH Terms] AND (“primary prevention” [MeSH Terms] OR “secondary prevention” [MeSH Terms]) “musculoskeletal pain” [MeSH Terms] AND “prevention”
Web of Science	TOPIC: (“back pain”) AND TOPIC: (“exercise” OR “exercise therapy”) AND TOPIC: (“clinical trial”) TOPIC: (“back pain”) AND TOPIC: (“health education” OR “cognitive behavioral therapy”) AND TOPIC: (“clinical trial”) TOPIC: (“back pain”) AND TOPIC: (“primary prevention” OR “secondary prevention”) AND TOPIC: (“clinical trial”) TOPIC: ("back pain") AND TOPIC: ("prevention") AND TOPIC: (“clinical trial”) TOPIC: (“neck pain”) AND TOPIC: (“exercise” OR “exercise therapy”) AND TOPIC: (“clinical trial”) TOPIC: (“neck pain”) AND TOPIC: (“health education” OR “cognitive behavioral therapy”) AND TOPIC: (“clinical trial”) TOPIC: (“neck pain”) AND TOPIC: (“primary prevention” OR “secondary prevention”) AND TOPIC: (“clinical trial”) TOPIC: (“neck pain”) AND TOPIC: ("prevention") AND TOPIC: (“clinical trial”) TOPIC: (“musculoskeletal pain”) AND TOPIC: (“exercise” OR “exercise therapy”) AND TOPIC: (“clinical trial”) TOPIC: (“musculoskeletal pain”) AND TOPIC: (“health education” OR “cognitive behavioral therapy”) AND TOPIC: (“clinical trial”) TOPIC: (“musculoskeletal pain”) AND TOPIC: (“primary prevention” OR “secondary prevention”) AND TOPIC: (“clinical trial”) TOPIC: (“musculoskeletal pain”) AND TOPIC: (“prevention”) AND TOPIC: (“clinical trial”)
Scopus	((TITLE-ABS-KEY (“back pain” AND TITLE-ABS-KEY (“exercise” OR “exercise therapy”) AND TITLE-ABS-KEY (“clinical trial”)) ((TITLE-ABS-KEY (“back pain” AND TITLE-ABS-KEY (“health education” OR “cognitive behavioral therapy”) AND TITLE-ABS-KEY (“clinical trial”)) ((TITLE-ABS-KEY (“back pain” AND TITLE-ABS-KEY (“primary prevention” OR “secondary prevention”) AND TITLE-ABS-KEY (“clinical trial”)) ((TITLE-ABS-KEY ("back pain" AND TITLE-ABS-KEY ("prevention") AND TITLE-ABS-KEY (“clinical trial”)) ((TITLE-ABS-KEY (“neck pain” AND TITLE-ABS-KEY (“exercise” OR “exercise therapy”) AND TITLE-ABS-KEY (“clinical trial”)) ((TITLE-ABS-KEY (“neck pain” AND TITLE-ABS-KEY (“health education” OR “cognitive behavioral therapy”) AND TITLE-ABS-KEY (“clinical trial”)) ((TITLE-ABS-KEY (“neck pain” AND TITLE-ABS-KEY (“primary prevention” OR “secondary prevention”) AND TITLE-ABS-KEY (“clinical trial”)) ((TITLE-ABS-KEY (“neck pain” AND TITLE-ABS-KEY ("prevention") AND TITLE-ABS-KEY (“clinical trial”)) ((TITLE-ABS-KEY (“musculoskeletal pain” AND TITLE-ABS-KEY (“exercise” OR “exercise therapy”) AND TITLE-ABS-KEY (“clinical trial”)) ((TITLE-ABS-KEY (“musculoskeletal pain” AND TITLE-ABS-KEY (“health education” OR “cognitive behavioral therapy”) AND TITLE-ABS-KEY (“clinical trial”)) ((TITLE-ABS-KEY (“musculoskeletal pain” AND TITLE-ABS-KEY (“primary prevention” OR “secondary prevention”) AND TITLE-ABS-KEY (“clinical trial”)) ((TITLE-ABS-KEY (“musculoskeletal pain” AND “TITLE-ABS-KEY (“prevention”) AND TITLE-ABS-KEY (“clinical trial”))
Medline	(MH “back pain”) AND (MH “exercise” OR MH “exercise therapy”) AND (MH “clinical trial”) (MH “back pain”) AND (MH “health education” OR MH “cognitive behavioral therapy”) AND (MH clinical trial”) (MH “back pain”) AND (MH “primary prevention” OR MH “secondary prevention”) AND (MH “clinical trial”) (MH “back pain") AND “prevention" AND (MH “clinical trial”) (MH “neck pain”) AND (MH exercise” OR MH “exercise therapy”) AND (MH “clinical trial”) (MH “neck pain”) AND (MH “health education” OR MH “cognitive behavioral therapy”) AND (MH “clinical trial”) (MH “neck pain”) AND (MH “primary prevention” OR MH “secondary prevention”) AND (MH “clinical trial”) (MH “neck pain”) AND “prevention" AND (MH “clinical trial)” (MH “musculoskeletal pain”) AND (MH “exercise” OR MH “exercise therapy”) AND (MH “clinical trial”) MH “musculoskeletal pain” AND (MH “health education” OR MH “cognitive behavioral therapy”) AND (MH “clinical trial”) MH “musculoskeletal pain” AND (MH “primary prevention” OR MH “secondary prevention”) AND (MH “clinical trial”) MH “musculoskeletal pain” AND “prevention” AND MH “clinical trial”

### Study selection

After removing duplicates, two reviewers (P.H.-L) and (J. L.-B.) independently screened articles for eligibility. In case of disagreement, both reviewers debated until an agreement was reached. The following inclusion criteria were applied for the selection of studies: (i) published in the last five years; (ii) ET plus HE was administered to the study sample; (iii) the sample consisted of participants with NBP; (iii) the research included a group that received UC, with neither exercise nor education. On the other hand, studies with the following characteristics were excluded from this review: (i) no quasi-experimental and observational studies; (ii) participants with specific causes of back pain; (iii) pregnant women; (iv) full text not available.

After screening the data, extracting, obtaining, and screening the titles and abstracts for inclusion criteria, the selected articles were obtained in full texts. Articles with titles and abstracts lacking sufficient information regarding the inclusion criteria were also obtained in full text. Full text articles were selected in case of compliance with inclusion criteria by the two reviewers using a data extraction form.

### Data extraction and quality assessment

The two reviewers independently extracted data from the included studies using a customized data extraction table developed in Microsoft Excel. In case of disagreement, both reviewers debated until an agreement was reached.

The following data from the included articles were selected for further analysis: demographic information (title, authors, journal, and year), characteristics of the sample (age, gender, inclusion and exclusion criteria, and number of participants), study-specific parameters (duration of the intervention, adverse events, methods of ET and HE) and results obtained (variables analyzed, instruments used and time of follow-up). Tables were used to describe both the studies’ characteristics and the extracted data.

The Jadad scale and PEDRO scale were used to assess the quality of the studies.

### Data synthesis and analysis

Tables were used to describe both the studies’ characteristics and the extracted data. When possible, the results were gathered based on type of intervention applied.

Standardized mean differences (SMD) and their 95% confidence interval (CI) were calculated as the between-group difference in means divided by the pooled standard deviation (SD) [39]. SMDs were interpreted using the following cut-off values: 0–0.2 (very small); 0.2–0.5 (small); 0.5–0.8 (moderate); and >0.8 (large) [40]. The same increments were used for negative values. The significance level was set to *p* < 0.05. The I^2^ statistic was used to determine the degree of heterogeneity, where the percentages quantified the magnitude of heterogeneity: 25% = low; 50% = medium; and 75% = high heterogeneity [41]. The analyses were performed with Comprehensive Meta-Analysis (CMA) V2 software (Biostat, NJ, USA).

## Results

### Study selection

**Figure 1.**
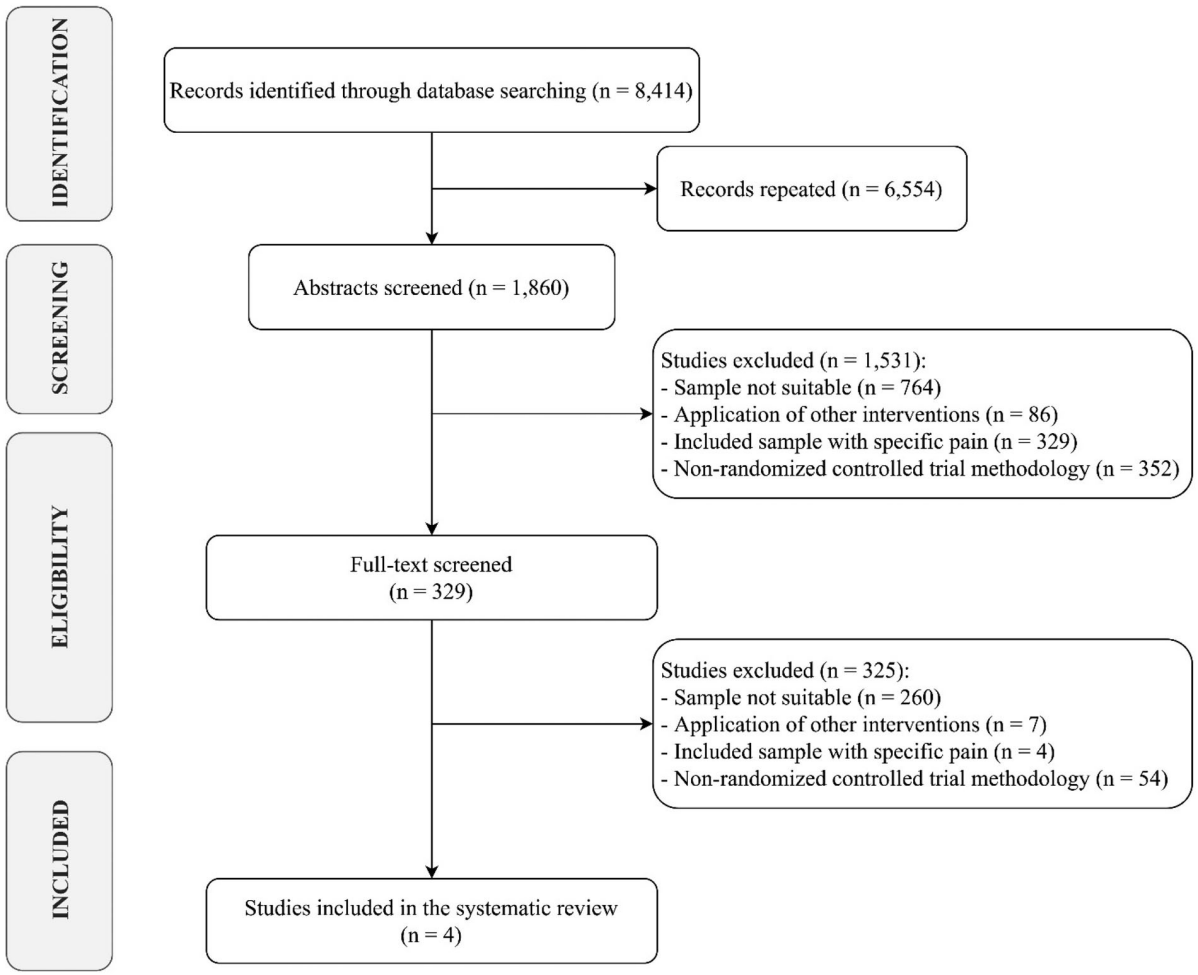
PRISMA flow diagram.

Out of 8414 search results, 1832 studies were considered eligible for inclusion after removing duplicates. Among the 1860 papers screened, 1531 were excluded after abstract and title screening. After the first reading of all candidate full texts, the Kappa score of reviewers 1 and 2 was 0.85 (i.e. almost perfect) [42]. All four full-text articles assessed for eligibility were finally included in the synthesis, as depicted by the PRISMA flowchart in Figure 1.

### Samples and risk of bias of included studies

The methodological quality of the studies was five or more points on the PEDRO scale and the average score was 6.5 points (Table 2). According to the PEDRO scale, the studies have a good methodological quality [43]. At the same time, on the JADAD scale, two works [44,45] were rated with tree points and the other two works [46,47] received four points. The most common methodological shortcoming was the absence of blinding [44,45] (Table 3).

**Table 2.** Risk of bias assessment using the Physiotherapy Evidence Database (PEDro) scale.

Author	1^a^	2	3	4	5	6	7	8	9	10	11	Score
Sandal et al. [41]	Yes	Yes	No	Yes	No	No	No	Yes	Yes	Yes	Yes	7
Antúnez et al. [45]	Yes	Yes	Yes	Yes	No	No	No	No	Yes	Yes	Yes	7
Turner et al. [44]	Yes	Yes	No	No	No	No	No	Yes	Yes	Yes	Yes	5
Cherkin et al. [46]	No	Yes	No	Yes	No	No	Yes	Yes	Yes	Yes	Yes	7

Criteria: (1) Eligibility criteria specified; (2) Subjects randomly allocated to groups; (3) Concealed allocation; (4) Groups were similar at baseline; (5) Blinding of all subjects; (6) Blinding of all therapists; (7) Blinding of all assessors; (8) Measures obtained from more than 85% of subjects allocated to groups; (9) Subjects received treatment or control condition as allocated, or intention-to-treat analysis; (10) Between-group statistical comparisons reported for at least one outcome; (11) Both point measures and measures of variability were reported. High, high risk of bias; low, low risk of bias. ^a^This item relates to external validity and therefore does not contribute to the total score.

**Table 3.** Baseline characteristic of patients and Jadad scale risk of bias.

Characteristic	**Sandal** et al. **[**41**]**	**Antúnez** et al. **[**45**]**	**Turner** et al. **[**44**]**	**Cherkin** et al. **[**46**]**
Sample (*n*)	461	90	342	342
Female (%)	55.3%	76.7%	65.7%	65.7%
Mean age (years)	47.5	38.3	49.3	49.3
Inclusion criteria	Age over 18 years, with NLBP within the preceding 8 weeks, scored 6 points or higher on the RMDQ in the region of Southern Denmark, had a smartphone, and had access to email.	NNP of less than one month of evolution with the aim of acting in acute/subacute states of this problem, autonomy to meet the demands of the study. Interruption of the pharmacological treatment prescribed or associated with the symptomatology of NNP.	Age between 20 and 70 years, NLBP for at least 3 months, patient-rated Pain during the previous wee*k* ≥ 4 (0 − 10 scale), and patient-rated pain interference with activities during the previous wee*k* ≥ 3 (0 − 10 scale).	Age between 20 and 70 years with NLBP that persisted at least 3 months.
Exclusion criteria	Inability to carry out the intervention, fibromyalgia, previous spinal surgery, current pregnancy, current participation in other NLBP-focused research, or an RMDQ score lower than 6 points at screening.	NNP with neurological involvement. Inflammatory, rheumatic and/or degenerative bone disease. Positive Jackson and Valsalva test.	Pregnancy, spine surgery in the previous 2 years, disability compensation, fibromyalgia or cancer, other major medical condition, plans to see a medical specialist for back pain, inability to read or speak English, and participation in a treatment for back pain in the past year.	Back pain associated with a specific diagnosis with compensation or litigation issues, difficulty participating, rated pain> =4 or pain interference with activities at less than 3 on 0- to 10-point scales.
Randomization	2	2	2	2
Blinding	1	0	0	1
Withdraw	1	1	1	1
Jadad’s score	4	3	3	4

NLBP: non-specific low back pain; RMDQ: Roland-Morris Disability Questionnaire; NNP: non-specific neck pain.

### Baseline characteristic of patients

A total of 1,235 patients took part in the included studies [44–47] (65.9% of whom are women). The mean age of the participants was 46 years. For more details, see Table 3. The participants only experienced adverse effects in one study [46], and these were discomfort, pain, or harm caused by the intervention. In two articles [44,45], the authors do not refer to the adverse effect.

### Interventions applied

The average number of sessions was 12, with 60–120 min being the most common range of session time [44–46]. Antúnez et al. [45] completed five sessions per week, while other two studies [44,46] attended one session per week. Sandal et al. [47] did not report the duration and number of sessions per week.

The ET performed in the four selected studies were yoga [44,46] and strengthening and stretching exercises [45,47]. On the other hand, the HE was focused on ergonomics [45], pain neuroscience education [47], and mindfulness and stress reduction techniques [44,46]. These interventions were supervised by physiotherapists [45], physicians [47] and psychologists [44,46] (Table 4).

**Table 4.** Characteristics of the included trials.

Characteristic	**Sandal** et al. **[**41**]**	**Antúnez** et al. **[**45**]**	**Turner** et al. **[**44**]**	**Cherkin** et al. **[**46**]**
Sample	461	90	342	342
Pain area	Lumbar	Cervical	Lumbar	Lumbar
Intervention	G1: HE + ET CG: UC	G1: HE + ET CG: UC	G1: HE G2: HE + ET CG: UC	G1: HE + ET G2: HE CG: UC
Adverse effects	No	*Not reported*	*Not reported*	Discomfort, pain or harm
Supervisor	Physician	Physiotherapist	Psychologist	Psychologist
Duration of intervention	9 weeks	3 weeks	8 weeks	8 weeks
Frequency of sessions (duration)	*Not reported*	5 x week (60′)	1 x week (120’)	1 x week (120’) G1: optional 6-hour retreat
Results identified	G1 significantly improved pain, disability, illness perception and perceived effect versus G2. Both groups improved fear and quality of life from baseline but not between groups.	Both groups significantly improved pain and disability from baseline. G1 significantly improved pain and disability versus G2.	G1 and G2 significantly improved pain catastrophizing, pain self-efficacy and pain acceptance versus G3. G2 obtained higher results than G1.	G1 and G2 significantly improved pain and disability versus G3 at 8, 26 and 52 weeks. G2 significantly improved depression and anxiety versus G1 and G3 at 8 and 26 weeks. G1 and G2 significantly improved the mental component of SF-12 versus G3 at 8 weeks. G2 significantly improved the mental component of SF-12 versus G3 at 26 weeks.

G1: Group 1; G2: Group 2; CG: Control group; HE: health education; ET: Exercise therapy; UC: Usual Medical Care.

### Meta-analysis results

**Figure 2.**
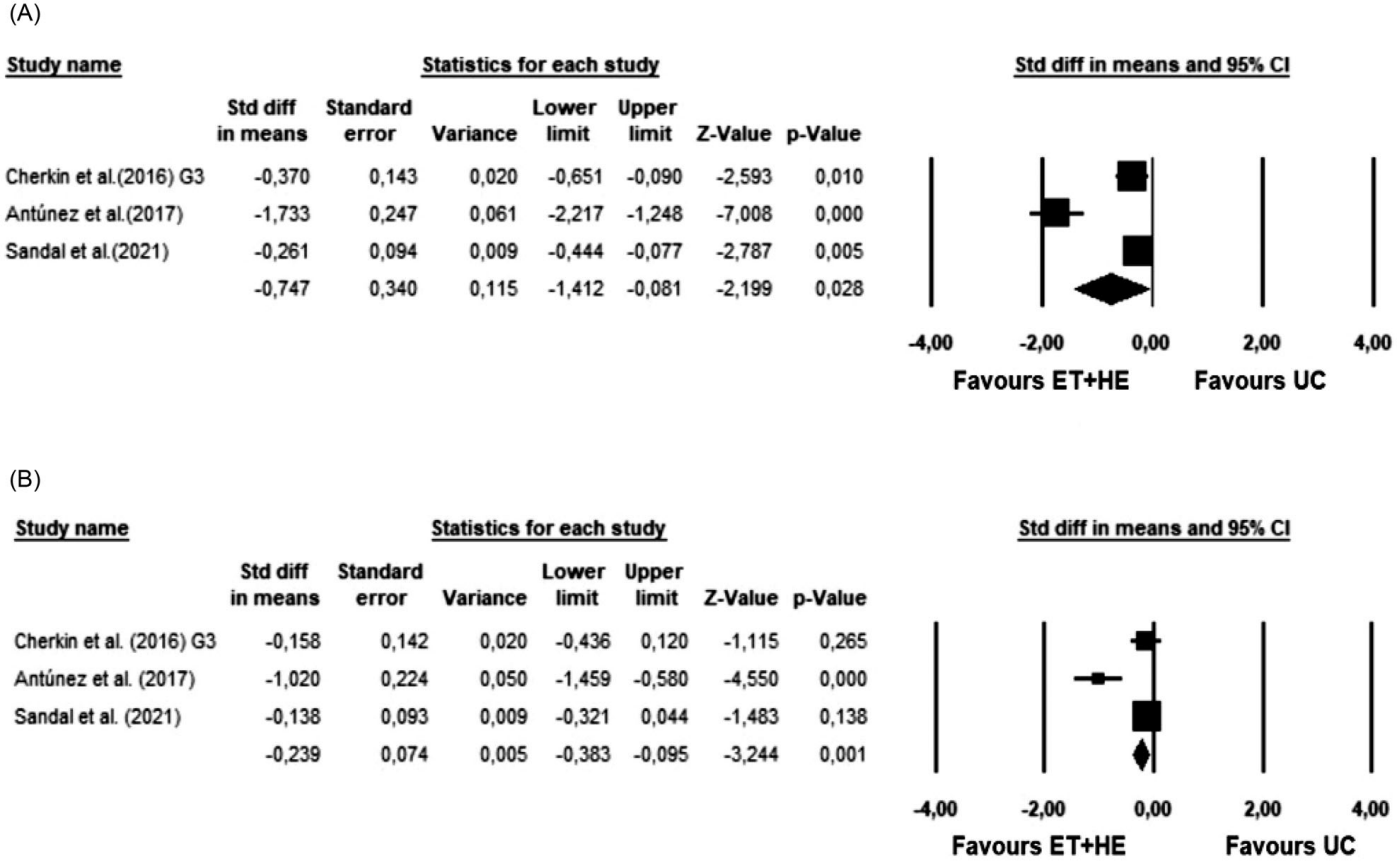
Forest plots of meta-analysis results. (A) Meta-analysis results for PAIN; (B) Meta-analysis results for DISABILITY. ET: Exercise Therapy; HE: Health Education; UC: Usual Medical Care.

Four studies included in this systematic review analyzed the pain variable, with a total sample size of 1,235 participants [44–47]. Three studies [45–47] were included in the meta-analysis. (*p* < 0.001, I^2^ = 93.6%). SMD effect size was found to be −0.75 (*p* = 0.03), with a variance of 0.115 (95% CI = −1.41 to −0.08). The forest plot is shown in Figure 2(A).

Three articles [36–38] with a total sample of 751 participants analysed the variable disability. All three studies were included in the meta-analysis (Figure 2(B)). The Q-test established heterogeneity across the studies and was high (*p* = 0.001, I^2^ = 85.3%). SMD effect size was found to be −0.24 (*p* = 0.001), with a variance of 0.005 (95% CI = −0.38 to −0.1).

Turner et al. [44] research is the only one that indirectly assesses kinesophobia. With a sample of 342 participants, they analyzed this variable. A significant post-intervention improvement was observed (*p* < 0.01), with a mean difference between groups of −3.3 and (95% CI −5.11 to −1.5).

## Discussion

The aim of this study was to determine whether the combination of ET and HE is more effective for the prevention of NBP than UC. The results suggest that there is a positive effect of combining ET and HE in the prevention of NBP. In addition, improvements were observed in other variables, such as disability and kinesophobia.

In the studies analyzed [45–47], the combination of ET plus HE obtained significant improvements on NBP compared to UC. Although it should be mentioned that the effect found in the meta-analysis is moderate. Different reviews confirm the benefits of exercise in non-specific low back pain [24–27] and in non-specific cervical pain [28–30,33]. In addition, two of these reviews compare the effect of ET versus UC [24,25], including several articles in which yoga is used, as in two of the studies [44,46] included in this review, or strengthening and flexibility exercises, as also occurs in these works [45,47].

In addition, Miyamoto et al. [34] conclude that exercise is cost efficient versus UC in the subacute and chronic treatment of non-specific low back pain. The results seem to indicate that theoretical-practical interventions have better results in the prevention of NBP than totally passive interventions included within UC [45–47], suggesting that exercise is cost efficient versus UC in the subacute and chronic treatment of low back NBP. This effect could be due to the multifactorial origin of NBP: some risk factors for NBP have a biophysical origin, such as a lack of strength or flexibility of the spinal musculature [12,48]. Other risk factors have a psychological origin, such as fear or stress, or even social factors such as false beliefs about NBP or work-related factors [17,49]. Therefore, the latest clinical guidelines on acute and chronic low back pain highlight exercise and education as key elements in clinical interventions [22].

The disability variable also showed a positive result in the meta-analysis [45–47]. These results are coherent, since disability is strongly related to pain, due to the close relationship between the physical and psychosocial components [50]. In the same line, two reviews conclude that ET produces an improvement in function in the lumbar [26] and cervical regions [28]. Kinesiophobia and catastrophism are two prognostic factors of clinical results in low back pain that are associated with disability [51]. Other authors have also found significant relationships between catastrophizing and the anxiety reaction to pain [52], as well as with the perception of pain intensity [53]. Turner et al. [44] observed that the combination of ET and HE is more effective than UC in reducing catastrophizing, and they also observed improvements in Pain Self-Efficacy. The International Association for the Study of Pain also establishes a relationship between fear-pain-knowledge, as they state that pain represents not only the sensation of physical harm, but also an emotional experience that can be influenced by other emotions, such as anxiety or fear of the unknown [54]. For all these reasons, the biopsychosocial approach is the current paradigm in the treatment and prevention of NBP [55].

Currently, there are reviews that analyse the effects of exercise applied in isolation in which a larger number of articles are analysed [25,33]. However, the novel objective of this review makes the inclusion of articles more demanding since only those interventions that combine HE and ET were included. Despite this, all the articles included in this review are randomized controlled trials with a high methodological quality and with a large number of participants overall. This makes it possible to draw a first conclusion while awaiting new studies that provide greater strength to this novel meta-analysis.

### Limitations

Among the limitations of the present investigation, the authors must acknowledge that they have not taken into account differentiated analyses by gender and age subgroups, nor have they included studies comparing the combination of ET and HE with UC in pregnant women. It is worth mentioning that, due to the high heterogeneity in the analyzed studies, it was not possible to establish which ET and HE interventions are the most effective, as well as the most appropriate frequency and duration of sessions. However, this is the first meta-analysis that analyzes the effects of the combination of exercise therapy, and HE compared to usual medical care in the prevention of LBP. In view of the above, further research is needed to compare the effects of different interventions with the aim of developing specific protocols for NBP prevention.

## Conclusions

Interventions combining ET and HE seem to have a greater preventive effect on NBP than UC. In addition, combining exercise with educational interventions has a higher improvement on disability and kinesophobia than UC.

The obtained results may help healthcare professionals to increase the effectiveness of their clinical interventions and thus reduce the severe socioeconomic impact caused by NBP worldwide.

## Author contributions

P. H.-L., R. L.-R., J. L.-B., J. L. G.-S. conceptualized and designed the study, drafted the initial manuscript, designed the data collection instruments, collected data, carried out the initial analyses, and critically reviewed the manuscript for important intellectual content. All authors have read and agreed to the published version of the manuscript.

## Data Availability

The dataset used and analyzed during the current study are available from the corresponding author.
